# Complete Genome of a *Methanosarcina mazei* Strain Isolated from Sediment Samples from an Amazonian Flooded Area

**DOI:** 10.1128/genomeA.00271-13

**Published:** 2013-05-23

**Authors:** Diego Assis das Graças, Rommel Thiago Jucá Ramos, Ana Carolina Vieira Araújo, Ramiro Zahlouth, Adriana Ribeiro Carneiro, Thiago Souza Lopes, Rafael Azevedo Baraúna, Vasco Azevedo, Maria Paula Cruz Schneider, Vivian Helena Pellizari, Artur Silva

**Affiliations:** Institute of Biological Sciences, Federal University of Pará, Belém, PA, Brazila; Institute of Oceanography, University of São Paulo, São Paulo, Brazilb; Institute of Biological Sciences, Federal University of Minas Gerais, Belo Horizonte, MG, Brazilc

## Abstract

*Methanosarcina mazei* is a strictly anaerobic methanogen from the *Methanosarcinales* order, which is known for its broad catabolic range among methanogens and is widespread throughout diverse environments. The draft genome of the strain presented here was cultivated from sediment samples collected from the Tucuruí hydroelectric power station reservoir.

## GENOME ANNOUNCEMENT

Methane is the second most important greenhouse gas, and approximately 80% is produced by biogenic sources (1). The only organisms able to produce methane are methanogenic archaea (2). Methanogens are found in several environments, such as rumen, rice fields, sea and freshwater sediments, and flooded areas (2). Although the methanogenesis pathway is well known, some aspects of the core genome, genome evolution, and shared genes are still unclear.

*Methanosarcina mazei* is a methanogenic archaeon capable of metabolizing several substrates, including CO_2_, acetate, and methylated compounds (2). This species is found in several environments and was detected in abundance in the reservoir of the fourth largest hydroelectric dam in the world (3). To characterize the genome of this species, a sediment sample from the Tucuruí hydropower station reservoir was inoculated in mineral media (4) supplemented with acetate and methanol and was maintained in an H_2_:CO_2_ (80:20) atmosphere to enrich and cultivate *M. mazei*. The enrichment was conducted at 30°C under standard anaerobic conditions (5). After several molecular and cellular analyses, such as transmission electron microscopy, denaturing high-performance liquid chromatography, 16S rRNA gene sequencing, and fluorescence *in situ* hybridization, total DNA was extracted from a nonpure culture of *M. mazei*, amplified using phi29 DNA polymerase (BioLabs), and used as a source template for genome sequencing.

The genome was first sequenced using a SOLiD System V3 with a mate-paired library, which yielded 24,405,103 and 24,399,268 reads (50 bp) for the R3 and F3 tags, respectively. A second round of sequencing was performed using the SOLiD 5500 XL platform with a mate-paired library, resulting in a total of 113,588,848 reads (60 bp) for each tag (F3 and R3). All reads were filtered by Quality Assessment software (6), whereby reads with an average quality score below Phred 20 were removed.

The reads were assembled by Velvet (7) and Edena (8), and the redundant sequences were removed by Simplifier (9), resulting in a total of 16,811 contigs. The scaffolds were produced by mapping these contigs against the *M. mazei* GO1 (AE008384) strain using BLAST (BLASTn) software. We used the Graphical contig Analyzer for All Sequencing Platforms software (G4ALL) to manually curate and generate the genome scaffold with gaps. Many of these gaps were closed using CLC Genomics Workbench software after recursive mapping of the contigs against the draft genome (10, 11). The complete genome sequence of *M. mazei* TUC01 contained 3,427,949 bp with a GC content of 42.5%. The genome was annotated by RAST (12), and 3,252 coding DNA sequences (CDS) were predicted.

This is the first archaeal genome from a Brazilian environment, and it provides biological information that will be helpful for more in-depth analyses and for obtaining more insight into the ecology and genome evolution of the *Methanosarcinales* order.

### Nucleotide sequence accession number.

The genome sequence obtained in this study has been deposited in the GenBank database under accession number CP004144.1.
